# Exploring the Potential of Humoral Immune Response to Commensal *Bifidobacterium* as a Biomarker for Human Health, including Both Malignant and Non-Malignant Diseases: A Perspective on Detection Strategies and Future Directions

**DOI:** 10.3390/biomedicines12040803

**Published:** 2024-04-04

**Authors:** Kyogo Itoh, Satoko Matsueda

**Affiliations:** Cancer Vaccine Center, Kurume University, Kurume 839-0863, Japan

**Keywords:** antibody, non-self-antigen, bifidobacterial, antigenic mimicry, tumor cell

## Abstract

In this comprehensive review, we explore the pivotal role of commensal *Bifidobacterium* (c-BIF) as potent non-self-antigens through antigenic mimicry, along with exploring the potential of humoral immune responses for both malignant and non-malignant disease. c-BIF, a predominant component of the human gut microbiome encompassing around 90% of the human genome, has emerged as a pivotal player in human biology. Over recent decades, there has been extensive research elucidating the intricate connections between c-BIF and various facets of human health, with particular emphasis on their groundbreaking impact on anti-cancer effects and the management of non-malignant diseases. The multifaceted role of c-BIF is explored, ranging from enhancing anti-tumor immunity to improving the efficacy of anti-cancer and anti-infectious disease strategies, and serving as predictive biomarkers for various diseases. Recent studies highlight not only c-BIF’s promotion of anti-tumor immunity but also their role in enhancing the efficacy of immune checkpoint inhibitors. The review emphasizes the promising avenue of manipulating the gut microbiota, particularly c-BIF, for modulating cancer immunotherapy with targeted effects on tumor cells while minimizing harm to normal tissue. In the context of infectious and inflammatory diseases, the crucial role of c-BIFs in the management of COVID-19 symptoms is examined, emphasizing their impact on the severity of and immune response to COVID-19. Furthermore, c-BIF exhibits preventive and therapeutic effects on Human Papillomaviruses (HPV) and shows promise in improving inflammatory bowel diseases. The potential application of c-BIF as a biomarker for immunotherapy is explored, with a specific emphasis on its predictive and prognostic value in cancer. Suggestions are made regarding the use of humoral immune responses to cytotoxic T lymphocyte (CTL) epitope peptides that share motifs with c-BIF, proposing them as potential markers for predicting overall survival in diverse cancer patients. In conclusion, c-BIF emerges as a crucial and multifaceted determinant of human health, across anti-tumor immunity to infectious and inflammatory disease management. The manipulation of c-BIF and gut microbiota presents a promising avenue for advancing therapeutic strategies, particularly in the realm of cancer immunotherapy. Additionally, this review highlights the significance of c-BIF as potent non-self-antigens via antigenic mimicry, emphasizing the importance of robust humoral immune responses against c-BIF for preventing various diseases, including inflammatory conditions. Elevated levels of circulating antibodies against c-BIF in healthy individuals may serve as potential indicators of lower risks for malignant and non-malignant diseases.

## 1. Introduction

*Bifidobacterias* are prevalent inhabitants of the human gut and have garnered attention for their substantial contribution to promoting health and well-being [1]. The genetic revolution of recent years has significantly broadened our understanding of the intricate relationship between these commensal microorganisms and their human hosts. Notably, it has become known that a remarkable 90% of the human genome is composed of genes originating from these commensal *Bifidobacterium*, often abbreviated as “c-BIF”. This revelation, a groundbreaking discovery presented by Collen [2], underscores the profound impact these microorganisms have on human biology.

Over the past decade, an exciting body of research has emerged, unveiling the previously hidden connections between c-BIF and various aspects of human health. One of the most striking revelations in this line of research has been the correlation between c-BIF and their ability to confer anti-cancer effects. Additionally, studies have shed light on the role of c-BIF in combating infectious diseases. These discoveries underscore the importance of understanding the intricate interplay between *Bifidobacterias* and the human body, as they have the potential to revolutionize the fields of cancer research, immunology, and the development of novel therapeutic strategies for infectious diseases. This burgeoning area of research is a testament to the vital role that these commensal microorganisms play in shaping human health and offers promising avenues for future medical interventions.

In this review, we discuss the pivotal role of c-BIF in enhancing anti-tumor immunity, improving the effectiveness against infectious diseases, and serving as a predictive biomarker for both malignant and non-malignant diseases, including inflammatory conditions.

## 2. Microbiome and Immunosurveillance in Human

### 2.1. How c-BIF Interact with Human Immune System

The intestinal mucosal epithelium comprises intestinal epithelial cells (IECs), intraepithelial lymphocytes (IELs), paneth, and goblet cells as specialized secretory epithelial cells. Lamina propria is a connective tissue located beneath the epithelium mainly composed of Peyer’s patches consisting of different immune cells, including innate lymphoid cells (ILCs), inducible natural killer (iNK) cells, T and B lymphocytes as well as microfold cells (M cells) [3]. Peyer’s patches (PPs) share the important function of bringing together antigens and rare antigen-specific lymphocytes to foster the induction of adaptive immune responses. Antigens are delivered to PPs by specialized microfold (M) epithelial cells, and they may be captured and presented by resident dendritic cells (DCs). In accordance with their state of chronic microbial antigen exposure, PPs exhibit continual germinal center (GC) activity. These GCs contribute to the generation of B cells and plasma cells producing somatically mutated gut antigen-specific IgA antibodies but have also been suggested to support the antigen-nonspecific diversification of the B cell repertoire [4]. Intestinal effector sites are characterized by the diffuse distribution of lymphocytes among non-immune cells and their matrix and include the intraepithelial (IEL) compartment and the lamina propria (LP). The composition of these effector sites demonstrates significant bias toward specific subsets of lymphocytes. Within the IEL compartment, the majority of T cells express CD8 [5]; these CD8+ T cells contribute to tissue homeostasis and epithelial repair through the production of antimicrobial factors and tissue repair factors in response to intestinal microbiota [6].

Given the intricate interplay between the immune system and gut microbiota and the beneficial influence of gut microbiota on immune development, bacterial-based therapies present a promising strategy for enhancing the clinical outcomes of cancer immunotherapy and other chemotherapy [3].

### 2.2. The Homology of c-BIF with Tumor Antigens and Their Biological Role 

Genes and coded peptides recognized by T cells were first determined using c-DNA expression methods in 1991 [7]. Since then, thousands of genes and their respective antibody-recognized tumor antigen peptides have been determined using SEREX methods [8,9]. Over the past 30 years, researchers have identified large numbers of genes and tumor antigens recognized by the mechanisms of specific immunity [7,8,9,10,11,12,13,14,15,16,17]. Those determinations have enabled us to better understand the nature of tumor-associated antigens (TAAs) recognized by the cellular and humoral immune system. 

The human immune system, particularly the leukocytes, patrols tissues to eliminate invading pathogens, dying/dead cells, and senescent cells [14]. These mechanisms include recognizing and destroying malignant cells that expresses TAAs. The mechanisms involved in those immune responses against many antigens are not yet fully understood, but the recent advances showed that c-BIF have been found to carry particularly potent non-self-antigens: namely, antibodies against c-BIF antigens have been suggested to play a pivotal role for protection against malignant cells and also various diseases via antigenic mimicry. Bessell et al. reported that T cells that are specific to an epitope SVYRYYGL (SVY), expressed in the commensal bacterium *Bifidobacterium breve* (*B. breve*), cross-react with a neoantigen, SIYRYYGL (SIY) [18]. These SVY-specific T cells recognized SIY-expressing melanoma cells in vivo and contributed to decreased tumor growth and extended survival. They also demonstrated that T cell clones recognize naturally processed cancer antigens, such as MART-1/Melan-A and MELOE-1 that are cross-reactive with microbial peptides [19]. Vétizou et al. revealed that T cell responses specific for *B. thetaiotaomicron* or *B. fragilis* were associated with the efficacy of immune checkpoint inhibitors, CTLA-4 blockade [20]. Other studies have identified an association of the commensal microbiome with anti-PD-1 monoclonal antibody efficacy in epithelial tumors and melanoma [21,22,23]. 

Overall, it has been demonstrated that T cells recognize microbial peptides and can target cancer cells and improve survival, while the commensal microbiome’s composition has been linked to the effectiveness of immune checkpoint inhibitors in various cancer types. These findings enhance our understanding of the complex interplay between the immune system and cancer, offering potential avenues for improved therapeutic strategies.

### 2.3. The Role of c-BIF for Anti-Tumor Immunity

A recent study reported that c-BIF not only promoted anti-tumor immunity but also enhanced the efficacy of immune checkpoint inhibitors. In a mouse cancer model, Sivan et al. reported that c-BIF promoted antitumor immunity and facilitates anti-programmed cell death protein 1 ligand 1 (PD-L1) efficacy by promoting the T cell infiltration of solid tumors [13]. They conducted a study comparing melanoma growth in mice with distinct commensal microbiota and reported observing variations in spontaneous antitumor immunity. These differences were nullified through co-housing or fecal transfer. An analysis of the 16S ribosomal RNA revealed an association between the antitumor effects and *Bifidobacterium*. Administering *Bifidobacterium* alone significantly enhanced tumor control, comparable to the efficacy of PD-L1 antibody therapy. Combining the two treatments nearly eradicated tumor outgrowth. The mechanism involved augmented dendritic cell function, leading to enhanced CD8+ T cell priming and accumulation in the tumor microenvironment. The findings suggest the potential of manipulating the microbiota to modulate cancer immunotherapy. Leveraging the host immune system emerges as a promising avenue in cancer therapeutics, offering targeted effects on tumor cells while minimizing harm to normal tissue. 

**Figure 1:**
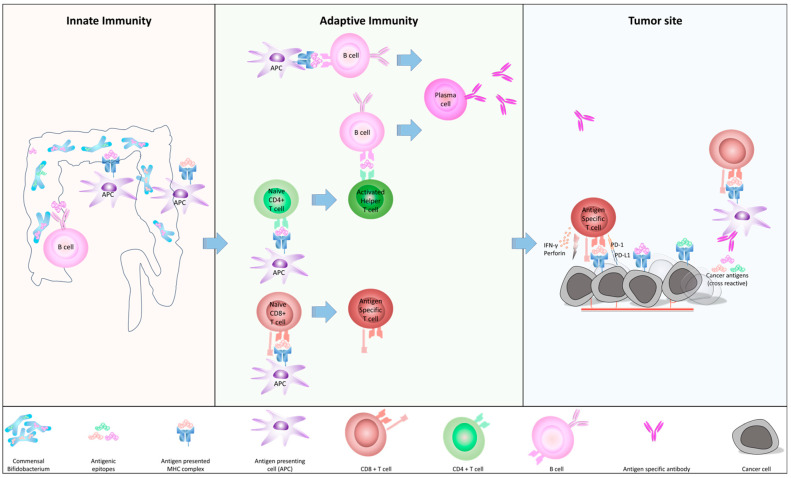
Interaction between c-BIF in gut and human immune system. Figure 1 left (innate immunity): The gut microbiome, including c-BIF is a source of non-self-antigens. They are processed by antigen presenting cells (APCs), the internalized antigen is digested into smaller peptides containing epitopes, and it is displayed by the major histocompatibility complex (MHC) as an antigen on the APC surface, such as dendritic cells. Figure 1, middle (adaptive immunity): It moves from the tissue to secondary lymphoid organs, such as lymph nodes, where it encounters and activates naïve CD4+ as well as CD8+ T cells. Naive CD4+ and CD8+ T cells gain antigen specificity against the epitopes that are presented on APC. Activated helper T (Th) cells then interact with the B cell through MHC and lead their maturation to plasma cells. B cells without MHC-II presentation of antigens to T cells, B-cell activation, and immunoglobulin class-switching can be facilitated by binding to activating transmembrane proteins BAFF and APRIL, which are secreted by myeloid cells like dendritic cells (DC) [24]. Figure 1, right (tumor site): Activated CD8+ antigen-specific T cells circulate in the bloodstream and reach the tumor site. Tumor antigens are processed and presented by cancer cells, which display high homology with c-BIF, are recognized by antigen-specific T cells, and destroyed. An antigen-specific antibody could bind epitopes to form an immune-complex and induce the maturation of APCs [25] in the tumor site. APCs then could activate naïve CD8+ cells to lead antigen-specific CD8+ killer T cells.

Sivan et al. emphasized that enthusiasm has been fueled by recent clinical success, particularly with antibodies that block immune inhibitory pathways, specifically CTLA-4 and the axis between PD-1 and its ligand 1 (PD-L1) [13]. This prompted Zitvogel et al. to hypothesize that c-BIF might have antigens sufficiently similar to human tumor antigens to be capable of eliciting tumor-specific T lymphocytes and antibodies recognizing future tumor cells via antigenic mimicry [14] (Figure 1). Vétizou et al. revealed that T cells that are specific for *B. thetaiotaomicron* or *B. fragilis* were associated with the efficacy of CTLA-4 blockade [20]. In the study, tumors in antibiotic-treated or germ-free mice did not respond to CTLA blockade. This defect was overcome by gavage with B. fragilis, by immunization with B. fragilis polysaccharides, or by adoptive transfer of B. fragilis-specific T cells. Study of fecal microbial transplantation (FMT) from humans to mice confirmed that treatment of melanoma patients with CTLA-4 blockade regulated the outgrowth of B. fragilis with anticancer properties. These results revealed a key role for Bacteroidales in the immunostimulatory effects of CTLA-4 blockade. Gopalakrishnan et al. reported that the gut microbiome modulates the response to anti-PD-1 immunotherapy in melanoma patients. The diversity and composition of the patient gut microbiome of responders exhibited significant differences compared to non-responders in melanoma patients who underwent anti-PD-1 immunotherapy [23]. They indicated that the diversity of the gut microbiome was significantly higher in responders compared to non-responders. 

In addition to immunotherapy, probiotic supplementation possibly enhances the antitumor effect of 5-Fluorouracil chemotherapy. Genaro et al. evaluated the effect of a probiotic on colorectal tumors in a rat model. The tumor-bearing rat group treated with 5-FU and a probiotic demonstrated an attenuated effect on the aggressiveness of colorectal tumors compared to the control group (*p* < 0.0003) [26,27].

The findings from these recent studies provide compelling evidence that the c-BIF plays a pivotal role in enhancing anti-tumor immunity and improving the efficacy of immune checkpoint inhibitors. These results underscore the potential for manipulating the gut microbiome to modulate cancer immunotherapy, offering a promising avenue for more effective and targeted cancer treatment.

## 3. The Importance of c-BIF in Infectious and Inflammatory Diseases

### 3.1. c-BIF Plays Pivotal Role in Managing Symptoms of COVID

Understanding the multidimensional role of c-BIF is crucial to enhancing diagnostic, preventive, disease control, and therapeutic potential in healthcare and improvement.

A diverse community of trillions of commensal bacteria inhabits mucosal and epidermal surfaces in humans and plays a significant role in the defense against pathogens, including respiratory pathogens [28]. The coronavirus disease 2019 (COVID-19) has spread all over the world since the pandemic started in 2019 [29]. It should be noted that people worldwide, whether vaccinated or unvaccinated, understand that the humoral immune responses against COVID-19 plays a pivotal role in protecting lives and preventing disease. Recent studies have demonstrated the involvement of gut microbiota in determining the severity of COVID-19 and dysfunctional immune responses in patients with COVID-19 [30,31,32]. Hazan et al. reported that lost microbes of COVID-19: *Bifidobacterium*, *Faecalibacterium* depletion, and decreased microbiome diversity associated with SARS-CoV-2 infection severity [31], reported the presence of the probiotic *Bifidobacterium* in the management of coronavirus. A theoretical basis [32] and Fujimoto et al. reported on the functional restoration of bacteriomes and viromes using FMT [33]. These results suggested that the depletion of *Bifidobacterium* generally led to reduced immune protection, allowing COVID-19 infection to become symptomatic. 

### 3.2. Preventive Therapeutic Effect of c-BIF on HPV 

Human Papillomaviruses (HPVs) are oncogenic viruses with a high degree of diversity and the most common sexually transmitted infectious disease (STD), leading to cervical, oropharyngeal, anal, penile, vaginal, and vulvar cancers [34]. An in vitro experiment exhibited that *Bifidobacterium adolescentis* SPM1005-A had antiviral activity through the suppression of E6 and E7 oncogene expression [35]. Abdolalipour et al. investigated the preventive–therapeutic effects of oral or intravenous administration of probiotic *Bifidobacterium bifidum* on immune response and tumor growth in a tumor mouse model with the TC-1 cell, such as the human papillomavirus (HPV)-related tumor, expressing HPV-16 E6/E7 oncogenes. Intravenous or oral administration of *Bifidobacterium bifidum* effectively induced antitumor immune responses and inhibits tumor growth in these mice. Interestingly, administering probiotic *Bifidobacterium bifidum* intravenously to mice with tumors triggers the activation of tumor-specific IL-12 and IFN-γ, as well as lymphocyte proliferation. This results in enhanced CD8+ cytolytic responses, effectively controlling tumor growth, in contrast to the oral route alone [36]. 

These studies highlight the potential of probiotic *Bifidobacterium* species, particularly *Bifidobacterium bifidum*, in modulating immune responses and suppressing the expression of HPV oncogenes, offering promise in the prevention and treatment of HPV-related tumors. Intravenous administration of this probiotic appears to be particularly effective in activating tumor-specific immune mechanisms, holding significant potential for controlling and eradicating tumor growth. It effectively conveys that the findings have opened up new possibilities for researching innovative therapies for HPV-associated malignancies.

### 3.3. The Therapeutic Effects of c-BIF as Probiotics for Inflammatory Diseases

Additionally, various inflammatory bowel diseases (IBD) including ulcerative colitis (UC) and Crohn’s disease showed improvement with supplementation probiotics, such as c-BIF [37]. Several studies have demonstrated a notable decrease in probiotics, specifically *Bifidobacterium* and *Lactobacillus*, as the gut microflora in patients with Inflammatory Bowel Disease (IBD) became more irregular [38]. Bozkurt et al. reported the efficacy of treating ulcerative colitis (UC) cases, demonstrating it through a single-dose intracolonic administration of a combination of *Bifidobacterium animalis subsp. lactis* and xylogluca. Administering two hundred billion colony-forming units (CFUs) of *B. animalis subsp. lactis* along with 4 g of xyloglucan in a single dose proved effective in promoting mucosal healing and resolving colonic symptoms in UC patients. [39]. Zhang C identified *Bifidobacterium longum FGDLZ8M1* as the most effective among *B. longum* strains (*B. longum FBJ20M1*, *B. longum FGDLZ8M1*, *B. longum FGSZY16M3*, and *B. longum FJSWXJ2M1*) in alleviating colitis. This strain demonstrated efficacy by reducing the expression of pro-inflammatory cytokines, restoring colon length, and maintaining mucosal integrity. They also revealed that the anti-inflammatory mechanisms of *B. longum* FGDLZ8M1 were related to the inhibition of NF-κB signaling [40].

The use of probiotics has shown promising results in improving inflammatory bowel diseases and offers a valuable therapeutic approach for managing these challenging conditions. Further research in this area holds significant promise for improving the well-being of IBD patients.

### 3.4. Microbial Signatures May Play a Critical Role for Immune-Related Adverse Events from Immunotherapy

Immune checkpoint inhibitors (ICIs) demonstrate high efficacy for the patients with advanced malignancies; however, they also can predispose patients to immune-related adverse events like immune-mediated colitis (IMC). Tayler et al. examined if microbiome alteration via fecal microbiota transplantation (FMT) is effective for refractory IMC. In the study, symptom improvement was observed in 83% (10 out of 12) of patients after Fecal Microbiota Transplantation (FMT). Additionally, 25% (3 out of 12) of patients required repeat FMT, with two showing no subsequent response. According to the study’s conclusion, 92% of participants achieved IMC clinical remission [41]. An analysis of patient stool samples using 16S rRNA sequencing revealed that those with complete responses (CR) exhibited significant increases in alpha diversity and higher abundances of *Collinsella* and *Bifidobacterium*. These microbial changes were particularly notable in FMT responders who were depleted in *Collinsella* and *Bifidobacterium* before undergoing FMT. Their study validates that FMT could be an effective treatment strategy for IMC and the microbial signatures that may play a critical role in FMT response [41]. Additional case reports also demonstrated improvement in symptoms for two patients with IMC, and the diversity and richness of microbiota were similar to healthy donor levels after FMT, which indicates that FMT contributed to recovery [42].

These promising results emphasize the need for continued research in this field and suggest FMT as a potential strategy to enhance patient outcomes in advanced malignancies treated with ICIs.

## 4. The Potential Use of c-BIF as Biomarkers and Treatment for Cancer Immunotherapies

### 4.1. Using c-BIF as Predictive and Prognostic Biomarkers for Colorectra Cancer (CRC)

Yamaoka et al. reported that the abundance of *Fusobacterium nucleatum* in colorectal cancer tissues correlated with tumor size, KRAS mutation, and was significantly associated with shorter overall survival times [43,44,45]. Likewise, a study conducted by Oh et al. revealed that disease-free survival was markedly superior in patients with high *F. nucleatum* levels compared to those with low/negative levels in non-sigmoid colon cancer (n = 219; log-rank *p* = 0.026). This suggests that the intratumoral *F. nucleatum* load is a potential prognostic factor in the non-MSI-high/non-sigmoid/non-rectal cancer subsets of stage II/III CRCs treated with oxaliplatin-based adjuvant chemotherapy [46]. Furthermore, according to the recent data reported by Hamada et al., *F. nucleatum* in CRCs differentially impacts tumor-infiltrating lymphocyte (TIL) density depending on the microsatellite instability (MSI) status [47,48]. Their high instability (MSI-H) refers to the high rate of somatic mutations accumulating in the region, and it is caused by an impaired DNA mismatch repair (MMR) [44]. MSI-H is an effective predictive biomarker to treat patients with CRC, particularly oxaliplatin or irinotecan-based therapy and recently immunotherapy [44,45,46,47,48,49,50]. Additionally, Rezasoltani et al. identified *Bifidobacterium* as a potential bacterial biomarker in CRC saliva samples, while *Fusobacterium*, *Dialister*, *Catonella*, *Tennerella*, *Eubacterium-brachy-group*, and *Fretibacterium* were ideal to distinguish healthy controls from CRC patients [51]. One potential topic for discussion could involve comparing the immunomodulatory effects of c-BIF alongside traditional probiotic supplementation in treatment “Probiotics” refer to live microorganisms, including certain strains of *Bifidobacterium*, that are ingested with the intention of providing health benefits [52,53]. Probiotics display a long history of safe utilization in both prevention and adjunctive therapy for various human diseases. Moreover, they show promise as potential agents for modulating the composition and function of the human gut microbiota in individuals with CRC [54,55]. There is emerging evidence suggesting that probiotics may play a role in minimizing the development and progression of colorectal cancer by mitigating the aggressiveness of tumors [56,57,58]. Some in vitro and in vivo studies have proposed potential mechanisms through which probiotics, including *L. lactis*, *L. plantarum*, *L. acidophilus*, and *B. animalis* subsp. *lactis*, contribute to the prevention of CRC [59,60,61,62]. These mechanisms encompass boosting the host immune response, triggering apoptosis, and inhibiting the tyrosine kinase signaling pathways [57,63]. Fahmy et al. demonstrated the oral administration of a probiotic, *B*. *longum*, to mice with CRC led to a significant reduction in the elevated expression of miR-155 as well as that of the onco-miR miR-21a. Notably, in both healthy and CRC-afflicted mice, *B. longum* treatment increased the levels of tumor-suppressing miR-145 and miR-15a. This probiotic intervention led to the downregulation of both NF-κB and miR-146a, which regulates the expressions of interleukin-1β (IL-1β) and interleukin-6 (IL-6) [64].

Together, *Bifidobacterium* and several other bacteria show promise as potential avenues for early detection and personalized treatment in colorectal cancer.

### 4.2. Humoral Immune Responses to Cytotoxic T lymphocyte (CTL) Epitope Peptides Shares Its Motifs with c-BIF Which Correlates to Overall Survival of Cancer

In our previous studies, we reported a compelling correlation between the levels of antibodies against CTL epitopes in pre-vaccination plasma and extended overall survival in multiple clinical studies for various types of cancer patients [17,25,65]. Furthermore, our investigations unveiled the presence of these humoral immune responses to CTL epitope peptides in the blood of both unvaccinated cancer patients and unvaccinated healthy donors, with notably higher detectable levels in younger generations [10,11,12,16,17,65] (Table 1). We also reported that immune boosting in both cellular and humoral responses with cancer vaccines using CTL epitope peptides was well correlated with overall survival with a hazard ratio of 0.2 (95% CI, 0.06–0.73; log-rank *p* = 0.0239) [48,66]. Despite the widespread presence of humoral responses against CTL epitope peptides from the lymphocyte-specific tyrosine protein kinase (Lck) antigen in both healthy donors and cancer patients, the functional implications of these antibodies have remained elusive. To address this gap in knowledge, we conducted investigations into the biological activity of the monoclonal antibody reactive to CTL epitope peptide of the Lck antigen at positions 486–494 (anti-Lck-486 mAb). Our findings revealed that this mAb induced the maturation of dendritic cells from murine bone marrow cells when forming immune complexes in vitro. Moreover, it effectively inhibited tumor growth, concomitant with the suppression of T regulatory cells at the tumor site. Notably, more potent tumor inhibition was observed with this when mAb was administered prior to peptide vaccination. These results underscore the significance of investigating the biological activity of anti-Lck peptide antibodies against CTL epitope peptides, which could hold promise for cancer immunotherapy [25]. 

Our exploration led us to hypothesize a potential homology between Lck peptides and a protein known as c-BIF. To evaluate this hypothesis, we conducted a BLAST search comparing Lck and c-BIF sequences. As expected, we found high homology between Lck peptide motifs and c-BIF sequences (representative c-BIF sequences and % identities are shown in Table 2). Remarkably, Lck motifs displayed homology with distinct types of c-BIF, indicating that antibodies against c-BIF may cross-react with Lck in the context of cancer (Table 2). These findings shed light on the ability of humoral immune responses to CTL epitope peptides to persist in the circulation, irrespective of vaccination status or disease, and suggest their potential as novel markers for predicting the overall survival of cancer patients [12,25].

In summary, probiotics are essential in maintaining a healthy balance of microorganisms in the human body, and their multifaceted roles extend from digestion to immune function, and various disease management strategies.

### 4.3. The Composition of the Gut Microbiome Is Recognized as a Key Factor in Influencing Effective Immuno-Oncology

The retrospective analysis conducted by Routy et al. discovered that primary resistance to immune checkpoint inhibitors can be attributed to abnormal gut microbiome composition [45]. FMT from cancer patients who responded to immune checkpoint inhibitors into germ-free or antibiotic-treated mice improved the antitumor effects of the PD-1 blockade, whereas fecal microbiota transplantation from nonresponding patients failed to do so. They then sought the impact of antibiotics on patients with advanced NSCLC (n = 140), RCC (n = 67), or urothelial carcinoma (n = 42) who received PD-1/PD-L1mAb after one or several prior therapies retrospectively. It revealed that progression-free survival (PFS) and overall survival (OS) were significantly shorter in the antibiotics-treated group. Interestingly, administering *A. muciniphila* orally following FMT using feces from non-responders reinstated the effectiveness of the PD-1 blockade. This restoration occurred in an interleukin-12-dependent manner and facilitated the increased recruitment of CCR9+CXCR3+CD4+ T lymphocytes into the tumor in a murine model [22]. Furthermore, Matson et al. reported that there was a significant association between commensal microbial composition and clinical response in metastatic melanoma patients. The findings indicated that responders had higher abundance of certain bacterial species, including *Bifidobacterium longum*, *Collinsella aerofaciens*, and Enterococcus faecium. Germ-free mice, when reconstituted with fecal material from responding patients, exhibited enhanced tumor control, increased tumor antigen-specific T cell responses, excluding regulatory T cells (Tregs: FoxP3+CD4+), and improved efficacy of anti-PD-L1 therapy. The findings imply that the therapeutic efficacy of anti–PD-1 therapy is linked to the composition of the commensal microbiota in patients [23].

The first inhuman trials to examine the clinical benefits of FMT were conducted by Davar et al. and Baruch et al. A phase 1 clinical trial (NCT03353402) was designed to evaluate the safety, feasibility, and impact of FMT and reinduction of anti–PD-1 immunotherapy in 10 patients with refractory metastatic melanoma [67]. In this study, stool samples were collected from two donor patients with metastatic melanoma who had been treated with anti-PD1 antibody and achieved a CR for at least one year. Ten recipients underwent an initial native microbiota depletion using antibiotics for 72 h. Patients received FMT using a colonoscopy and were administered oral stool capsules followed by reinduction of anti–PD-1 therapy. One CR and two PR were observed in ten patients. Notably, immunohistochemical (IHC) revealed that FMT treatment was associated with favorable changes, such as increased CD68+ APCs in gut lamina propria and CD8+ cytotoxic T cells in the tumor microenvironment. Clinical responses were observed in three patients (1 CR and 2 PR). Two out of the three who achieved clinical responses demonstrated pseudoprogression, which is an initial increase in metastases size followed by regression. The increase is attributed to the migration of antitumoral immune cells into the tumor site, rather than to the growth of tumor cells [68]. Considering safety, one patient experienced an FMT-related adverse event (AE), which was mild bloating. Grade 1 immune-related adverse events (irAEs), arthralgia (30%), rash (10), myalgia (10%), fatigue (10%), chills (10%), hepatobiliary disorder (10%), and ad vitiligo (10%), were observed [67]. Similarly, 16 melanoma patients who had no prior response to anti–PD-1 alone or in combination with CTLA-4 or investigational agents were recruited to the clinical trial (NCT03341143) [69]. In the trial, they collected stool samples from seven donors with advanced or metastatic melanoma treated with anti-PD-1, four of whom exhibited a complete response (CR) and three a partial response (PR), with a median progression-free survival (PFS) of 56 months (range: 45 to 70 months). The sampled stools were extensively studied to eliminate the possibility of transmitting infectious agents. The single donor-derived FMT was administered along with anti-PD-1 antibody to 16 melanoma patients. The objective response rate (ORR) was 20% (one CR and two PR among fifteen evaluated patients) and three out of fifteen patients (20%) showed durable stable disease (SD) lasting over 12 months. All patients experienced at least one AE (Grade 1, 72.9%; Grade 2, 20.0%). The frequently observed Grade 1 AEs were fatigue (9.7%), hyponatremia (16.1), and bloating (22.6%). Hypothyroidism occurred in three patients (17.6%) of nine and it was easily managed with hormone replacement. Three patients experienced Grade 3 AEs. One of the patients who experienced Grade 3 AEs experienced peripheral motor neuropathy and required hospitalization. However, it was resolved with intravenous immunoglobulin and corticosteroids and no further sequelae upon reinstitution of anti-PD1 therapy [69].

The observed significant association between gut microbiome composition and clinical response underscores the importance of comprehending and leveraging the microbiome in personalized cancer treatment strategies. These findings offer substantial promise for the advancement of cancer therapeutics and the enhancement of clinical outcomes. 

### 4.4. Exploring Potential Obstacles and Limitations in Immunotherapies Utilizing Microbiome Composition

FMT is a medical procedure that involves transferring fecal material from a donor (either healthy or patient who achieved clinical responses) to a recipient in order to restore a balance in gut microorganisms. While FMT has shown remarkable success in treating certain conditions, it also has limitations and considerations [70]:

(a) Safety Concerns:

FMT is generally considered safe and feasible, but there are potential risks of transferring infectious agents or pathogens from the donor to the recipient. Screening donors thoroughly is crucial to minimize these risks. For example, an extended spectrum beta-lactamase (ESBL) producing *E. coli* was identified as it originated from donated stool. This resulted in infections in patient’s bloodstreams, and unfortunately one patient died [71]. The FDA was notified to implement requirements on donor screening, to address risk factors for colonization with multi-drug-resistant organisms (MDROs), such as ESBL, vancomycin-resistant enterococci (VRE), carbapenem-resistant Enterobacteriaceae (CRE), and methicillin-resistant Staphylococcus aureus (MRSA) [72].

(b) Regulatory Challenges:

FMT is not uniformly regulated [73], and regulatory frameworks may vary across regions—FMT is considered a medical product in the UK, while it is considered an investigational biological product in the USA and Canada [70]. This can lead to challenges in ensuring standardized protocols and donor screening practices.

(c) Limited Understanding of Microbiome:

The human gut microbiome is complex and not fully understood yet, and it is possibly donor dependent even if a donor achieved CR [67]. While FMT can effectively introduce diverse microbial communities, the long-term effects and specific mechanisms are still under investigation.

(d) Treatment Specificity:

FMT has primarily been studied and proven effective for certain gastrointestinal conditions, and its efficacy for other disorders is less clear. It may not be a universally applicable treatment.

(e) Ethical and Psychological Factors:

Acceptance of FMT can be influenced by cultural, ethical, and psychological factors. Some patients and healthcare providers may have reservations about the concept of fecal transplantation.

(f) Standardization and Accessibility:

Standardized protocols for donor screening, preparation of fecal material, and the administration route of FMT are crucial. Lack of standardization may lead to inconsistent results and safety concerns [70]. FMT can be a costly procedure, and accessibility may be limited, especially in certain regions or healthcare settings. Cost and availability may affect its widespread adoption.

(g) Potential Long-Term Risks:

The long-term effects and risks associated with FMT are not fully investigated yet. Monitoring and research are ongoing to better understand any potential long-term consequences [74].

## 5. Conclusions

We explore the intriguing role of commensal *Bifidobacterium* (c-BIF) in the context of humoral immunity and its potential significance in preventing both malignant and non-malignant diseases. Humoral immunity is an essential component of the immune system that primarily involves the production of antibodies by B cells to combat foreign invaders or pathogens, such as bacteria, viruses, and malignant cells.

The central focus of this research is to investigate whether c-BIF, which are commensal bacteria residing in the human gut, can function as potent non-self-antigens through a mechanism called antigenic mimicry. It effectively describes antigenic mimicry as a phenomenon where the immune system mistakenly identifies a non-self-antigen as a threat, leading to an immune response. Additionally, it correctly notes that humoral immunity is typically designed to recognize and eliminate non-self-antigens while preserving self-antigens for the maintenance of a healthy state. This distinction is vital to prevent autoimmune reactions where the immune system attacks the body’s own tissues.

It suggests that c-BIF may contain non-self-antigens that resemble those found in malignant cells. This similarity could lead to the production of antibodies specifically targeting these c-BIF-derived antigens. These antibodies, it is hypothesized, might also provide protection against malignant cells through antigenic mimicry. The immune system might be “tricked” into attacking malignant cells due to their resemblance to c-BIF antigens, which would be beneficial in preventing the development of cancer.

The findings and implications presented in this review indicate that the humoral immune responses against c-BIF could be a crucial factor in preventing both malignant and non-malignant diseases. In other words, a robust and long-lasting immune response against c-BIF may contribute significantly to maintaining overall health. As a result, it is suggested that individuals with higher levels of circulating antibodies against c-BIF, particularly in healthy subjects, might have a lower risk of developing various inflammatory diseases, which are both malignant and non-malignant in nature.

In summary, this manuscript provides valuable insights into the potential role of c-BIF as potent non-self-antigens through antigenic mimicry, which could have significant implications for understanding and enhancing the body’s defenses against both cancer and other inflammatory diseases. Further research in this area may open doors to novel strategies for disease prevention and immune modulation.

## Figures and Tables

**Table 1:** Assessment of immunoglobulins reactive to each of the 31 different CTL epitopes in plasma or sera from healthy donors or patients.

Peptide Name	Original Protein	Position	Sequence	HLA-IA Restriction	Positive/Negative							
					Healthy ^(a)^ (n = 66)	HCV ^(b)^ (n = 20)	Flu ^(c)^ (n = 20)	RA ^(d)^ (n = 20)	IgA Nephropathy ^(e)^ (n = 20)	Leukemia ^(f)^ (n = 26)	Liver Cancer ^(g)^ (n = 41)	Solid Cancer ^(h)^ (n = 2588)
CypB-129	Cyclophilin B	129–138	KLKHYGPGWV	A2/A3 supertype	37/29	15/5	16/4	18/2	13/7	12/14	36/5	1888/700
Lck-246	p56 lck	246–254	KLVERLGAA	A2	46/20	16/4	17/3	17/3	16/4	11/15	35/6	1815/773
Lck-422	p56 lck	422–430	DVWSFGILL	A2/A3 supertype	19/47	6/14	11/9	8/12	7/13	5/21	13/28	316/2272
ppMAPkkk-432	ppMAPkkk	432–440	DLLSHAFFA	A2/A26	27/39	10/10	10/10	10/10	5/15	9/17	26/15	1308/1280
WHSC2-103	WHSC2	103–111	ASLDSDPWV	A2/A26/A3 supertype	64/2	20/0	20/0	20/0	19/1	24/2	41/0	1673/915
HNRPL-501	HNRPL	501–510	NVLHFFNAPL	A2/A26	15/51	13/7	10/10	14/6	4/16	5/21	27/14	1189/1399
UBE2V-43	UBE2V	43–51	RLQEWCSVI	A2	57/9	16/4	20/0	20/0	20/0	19/7	39/2	1238/1350
UBE2V-85	UBE2V	85–93	LIADFLSGL	A2	7/59	1/19	3/17	5/15	1/19	3/23	11/30	362/2226
WHSC2-141	WHSC2	141–149	ILGELREKV	A2	53/13	17/3	18/2	18/2	16/4	8/18	37/4	1908/680
HNRPL-140	HNRPL	140–148	ALVEFEDVL	A2	52/14	17/3	18/2	19/1	16/4	10/16	39/2	1295/1293
SART3-302	SART3	302–310	LLQAEAPRL	A2	45/21	16/4	18/2	14/6	14/6	12/14	24/17	1640/948
SART3-309	SART3	309–317	RLAEYQAYI	A2	48/18	16/4	18/2	19/1	15/5	15/11	41/0	1921/667
SART2-93	SART2	93–101	DYSARWNEI	A24	64/2	20/0	20/0	20/0	20/0	25/1	41/0	2248/340
SART3-109	SART3	109–118	VYDYNCHVDL	A24/A26/A3 supertype	15/51	6/14	7/13	7/13	3/17	6/20	33/8	1206/1382
Lck-208	p56 lck	208–216	HYTNASDGL	A24	31/35	11/9	13/7	9/11	7/13	4/22	22/19	560/2028
PAP-213	PAP	213–221	LYCESVHNF	A24	54/12	16/4	17/3	19/1	18/2	22/4	39/2	1422/1166
PSA-248	PSA	248–257	HYRKWIKDTI	A24	51/15	15/5	19/1	20/0	18/2	21/5	40/1	1286/1302
EGFR-800	EGF-R	800–809	DYVREHKDNI	A24	59/7	18/2	19/1	20/0	18/2	19/7	40/1	1542/1046
MRP3-503	MRP3	503–511	LYAWEPSFL	A24	3/63	1/19	2/18	2/18	1/19	2/24	4/37	467/2121
MRP3-1293	MRP3	1293–1302	NYSVRYRPGL	A24	59/7	18/2	20/0	19/1	16/4	19/7	41/0	1558/1030
SART2-161	SART2	161–169	AYDFLYNYL	A24	13/53	2/18	2/18	7/13	1/19	7/19	18/23	947/1641
Lck-486	p56 lck	486–494	TFDYLRSVL	A24	53/13	20/0	18/2	20/0	19/1	17/9	38/3	2184/404
Lck-488	p56 lck	488–497	DYLRSVLEDF	A24	64/2	20/0	20/0	20/0	20/0	25/1	41/0	2147/441
PSMA-624	PSMA	624–632	TYSVSFDSL	A24	41/25	20/0	14/6	17/3	8/12	11/15	38/3	950/1638
EZH2-735	EZH2	735–743	KYVGIEREM	A24	15/51	7/13	7/13	9/11	3/17	3/23	17/24	558/2030
PTHrP-102	PTHrP	102–111	RYLTQETNKV	A24	35/31	14/6	17/3	15/5	16/4	5/21	32/9	886/1702
SART3-511	SART3	511–519	WLEYYNLER	A3 supertype	60/6	17/3	20/0	20/0	20/0	21/5	40/1	1967/621
SART3-734	SART3	734–742	QIRPIFSNR	A3 supertype	66/0	20/0	20/0	20/0	20/0	25/1	41/0	2041/547
Lck-90	p56 lck	90–99	ILEQSGEWWK	A3 supertype	63/3	19/1	20/0	20/0	20/0	23/3	41/0	2087/501
Lck-449	p56 lck	449–458	VIQNLERGYR	A3 supertype	52/14	19/1	18/2	20/0	18/2	14/12	39/2	1406/1182
PAP-248	PAP	248–257	GIHKQKEKSR	A3 supertype	49/17	17/3	16/4	14/6	14/6	5/21	22/19	2035/553

Adopted and modified from Matsueda et al. [10] and Suekane et al. [11]. Plasma or serum were collected from ^(a)^ healthy donors: 15–64 years old, ^(b)^ patients with hepatitis C virus, ^(c)^ patients with influenza, ^(d)^ patients with rheumatoid arthritis, ^(e)^ patients with IgA nephropathy, ^(f)^ patients with leukemia, ^(g)^ patients with liver cancer who were not enrolled to cancer vaccine trials, and ^(h)^ patients with lung cancer (n = 399), prostate cancer (n = 354), colon cancer (n = 344), pancreatic cancer (n = 290), gastric cancer (n = 200), breast cancer (n = 183), and other types of cancer (n = 818) who are enrolled in cancer vaccine trials.

**Table 2:** BLAST search result between human tyrosine-protein kinase Lck and c-BIF.

Query	Subject	Position	Sequence	Identities (%)
Lck-90		90–99	ILEQSGEWWK	-
	*Bifidobacterium animalis*	497–504	*LQQSDEWW*	6/8 (75%)
	*Bifidobacterium longum*	480–487	IFEQNGEW**	6/8 (75%)
		924–932	*LEQSGDDEW	7/9 (78%)
		953–961	*LEQSGDDEW	7/9 (78%)
	*Bifidobacterium* sp.	2625–2630	***EQSGEW	6/6 (100%)
Lck-208		208–216	HYTNASDGL	-
	*Bifidobacterium bifidum*	270–277	HYTSATDG*	6/8 (75%)
	*Bifidobacterium castoris*	242–248	**TNASDGL	7/7 (100%)
		207–213	**TNASDGL	7/7 (100%)
	*Bifidobacterium dentium*	607–615	HYSDVSDGL	6/9 (67%)
	*Bifidobacterium italicum*	301–308	*YQNASDGL	7/8 (88%)
Lck-246		246–254	KLVERLGAA	-
	*Bifidobacterium avesanii*	363–369	KLVERLG**	7/7 (100%)
	*Bifidobacterium asteroides*	181–188	MVERLGAA	7/8 (88%)
	*Bifidobacterium breve*	471–478	KLVERFGA*	7/8 (88%)
	*Bifidobacterium longum*	300–307	*LVERLDAA	7/8 (88%)
		292–299	*LVERLDAA	7/8 (88%)
Lck-422		422–430	DVWSFGILL	-
	*Bifidobacterium crudilactis*	312–318	DVWSFAI**	6/7 (86%)
	*Bifidobacterium longum*	350–356	**WSIGILL	6/7 (86%)
		144–150	DVWNYGI	5/7 (71%)
	*Bifidobacterium breve*	178–184	DVWNYGI	5/7 (71%)
	*Bifidobacterium lemurum*	79–87	*VWKFGLILL	7/9 (78%)
Lck-449		449–458	VIQNLERGYR	
	*Bifidobacterium felsineum*	1076–1082	*IQNLERG**	7/7 (100%)
	*Bifidobacterium breve*	54–61	VIENL**GYR	7/10 (70%)
	*Bifidobacterium cebidarum*	741–747	*IQNLERG**	7/7 (100%)
	*Bifidobacterium pseudocatenulatum*	134–141	*INNLERGY	7/8 (88%)
	*Bifidobacterium coryneforme*	437–444	*QALDRGYR	6/8 (75%)
Lck-486		486–494	TFDYLRSVL	-
	*Bifidobacterium pullorum*	149–156	TFDYIRSV*	7/8 (88%)
	*Bifidobacterium longum*	318–323	*FDYLRS**	6/6 (100%)
		372–378	*FDYLQSV*	6/7 (80%)
	*Bifidobacterium breve*	12–18	**DYLRSVL	7/7 (100%)
	*Bifidobacterium animalis*	120–125	***YLRSVL	6/6 (100%)
Lck-488		488–497	DYLRSVLEDF	-
	*Bifidobacterium breve*	12–18	DYLRSVL***	7/7 (100%)
	*Bifidobacterium pseudolongum*	322–327	DYLRSV****	6/6 (100%)
	*Bifidobacterium longum*	132–139	**LRSILNDF	6/8 (75%)
	*Bifidobacterium animalis*	120–125	*YLRSVL***	6/6 (100%)
	*Bifidobacterium adolescentis*	76–84	*YLRSIPENF	6/9 (67%)

BLAST programs provided by NCBI for aligning query sequences against those present in a selected target database were used for this analysis. (https://blast.ncbi.nlm.nih.gov/Blast.cgi?PROGRAM=blastp&PAGE_TYPE=BlastSearch&LINK_LOC=blasthome, accessed on 30 August 2023). A * indicates a missing amino acid in the sequence compared to a query.
